# Supplementary material for: Porosity Tunable Poly(Lactic Acid)-Based Composite Gel Polymer Electrolyte with High Electrolyte Uptake for Quasi-Solid-State Supercapacitors
Source: Polymers (Basel). 2022 May 4;14(9):1881. doi: 10.3390/polym14091881 (PMC9105037; doi:10.3390/polym14091881)
Supplement: Supplementary file 1 [file polymers-14-01881-s001.zip › polymers-1706239-supplementary.pdf]

Supplementary Materials: Porosity Tunable Poly(Lactic Acid)-Based Composite Gel Polymer Electrolyte with High Electrolyte Uptake for Quasi-Solid-State Supercapacitors

Chao Yang [1,†], Yuge Bai [1,†], Huan Xu [3], Manni Li [1], Zhi Cong [1], Hongjie Li [1], Weimeng Chen [1], Bin Zhao [1], and Xiaogang Han [1,2*]

[1] State Key Laboratory of Electrical Insulation and Power Equipment, School of Electrical Engineering, Xi'an Jiaotong University, Xi'an 710049, Shaanxi, China; yc2018@stu.xjtu.edu.cn (C.Y.); baiyuge218@stu.xjtu.edu.cn (Y.B.); lmn4118004119@stu.xjtu.edu.cn (M.L.); jycz1997@stu.xjtu.edu.cn (Z.C.); lhj1124@stu.xjtu.edu.cn (H.L.); m6a8x3cwm@stu.xjtu.edu.cn (W.C.); and zhaobin87@xjtu.edu.cn (B.Z.)

[2] Key Laboratory of Smart Grid of Shaanxi Province, Xi'an 710049, Shaanxi, China

[3] School of Materials Science and Physics, China University of Mining and Technology, Xuzhou 221116, Jiangsu, China; hihuan@cumt.edu.cn

* Correspondence: xiaogang.han@xjtu.edu.cn

† These authors contributed equally.

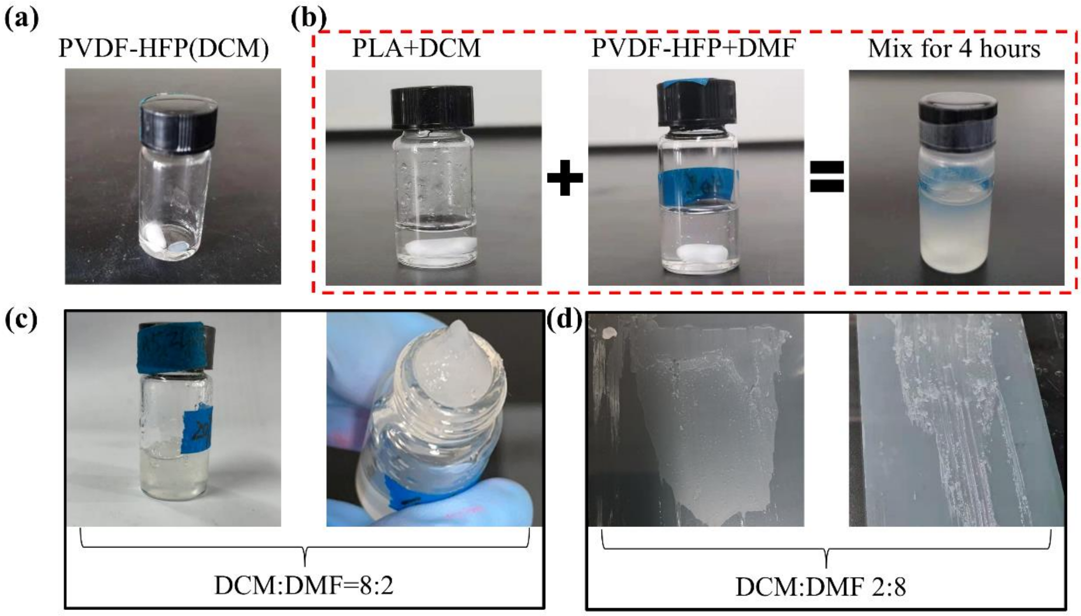

Figure S1. Experimental process and selection of solvent ratio.

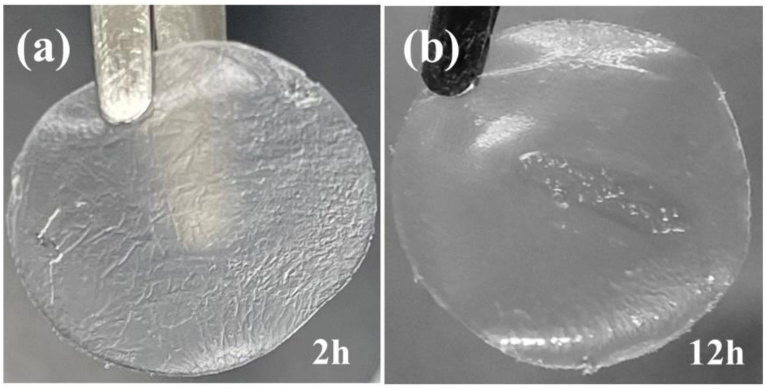

Figure S2. Morphologies of pure PLA samples.

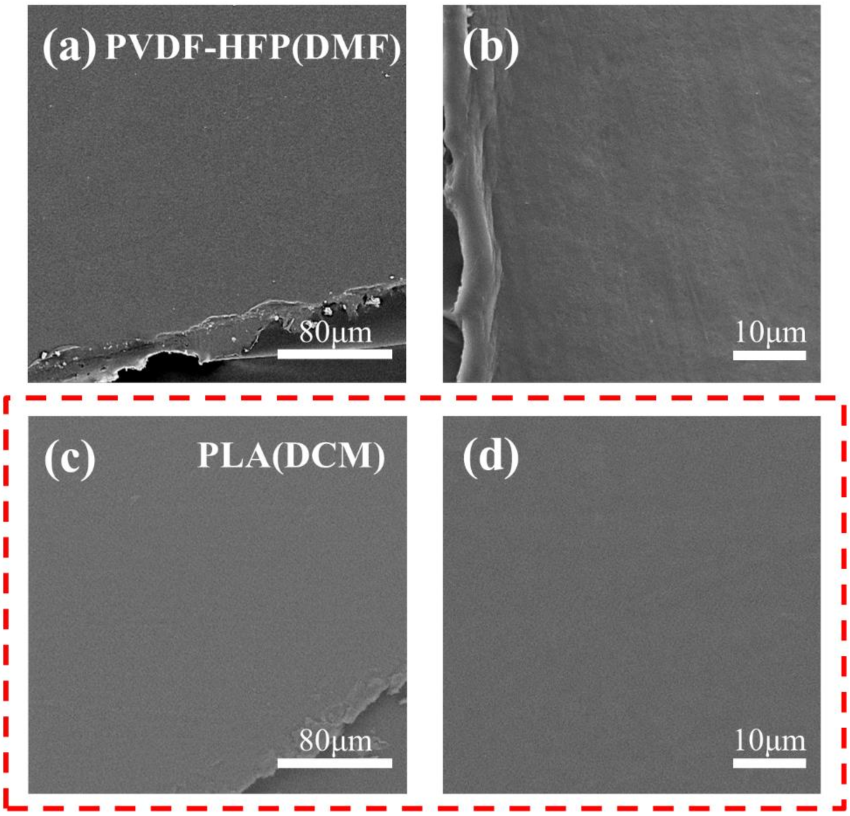

Figure S3. Micro morphologies of pure PVDF-HFP and pure PLA.

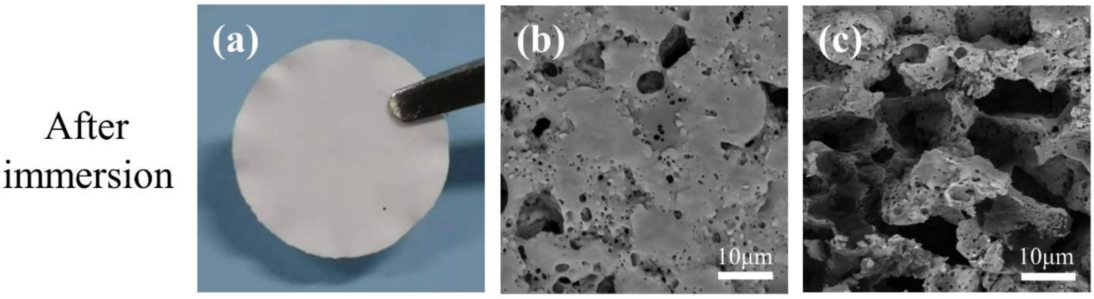

Figure S4. Optical and micro morphologies of optimized sample at the condition of just immersed.

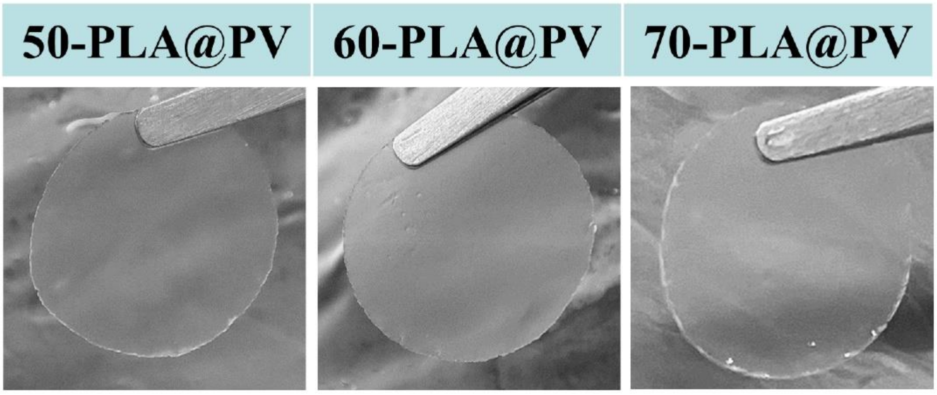

Figure S5. The digital images of PLA@PV based porous gel electrolyte.

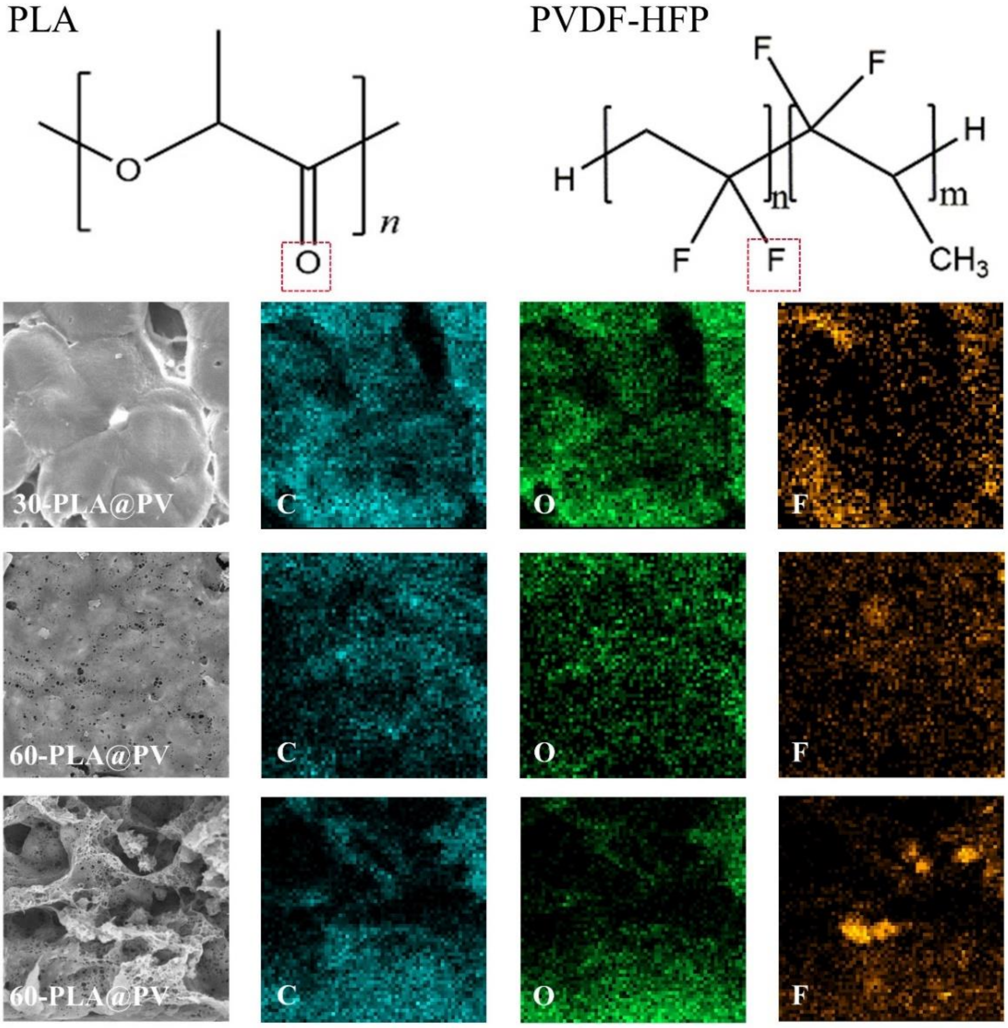

Figure S6. EDX mapping of 30-PLA@PV and 60-PLA@PV.

The EDX mapping of 60-PLA@PV are analyzed to identify the selective positioning of PLA and PVDF-HFP with characteristic elements O and F, respectively. As shown in Figure 3d and Figure 3i, the images reveal the uniform distribution of O and F on the membrane surface and cross section. It is apparent that PVDF-HFP is evenly distributed around the PLA matrix to ensure the structural stability of the composite membrane.

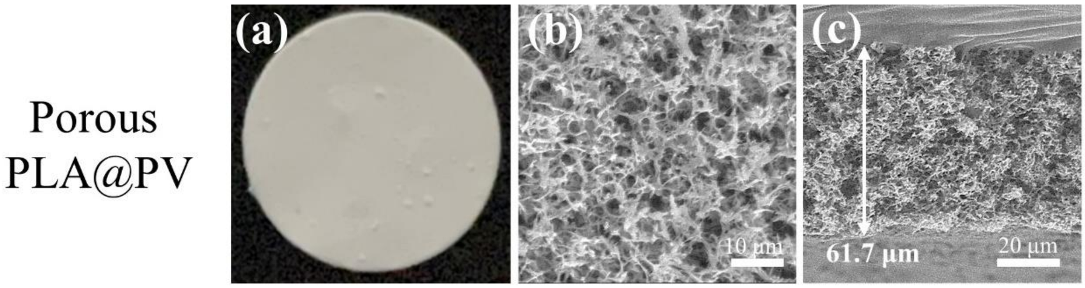

Figure S7. Optical and micro morphologies of pure porous PLA membrane.

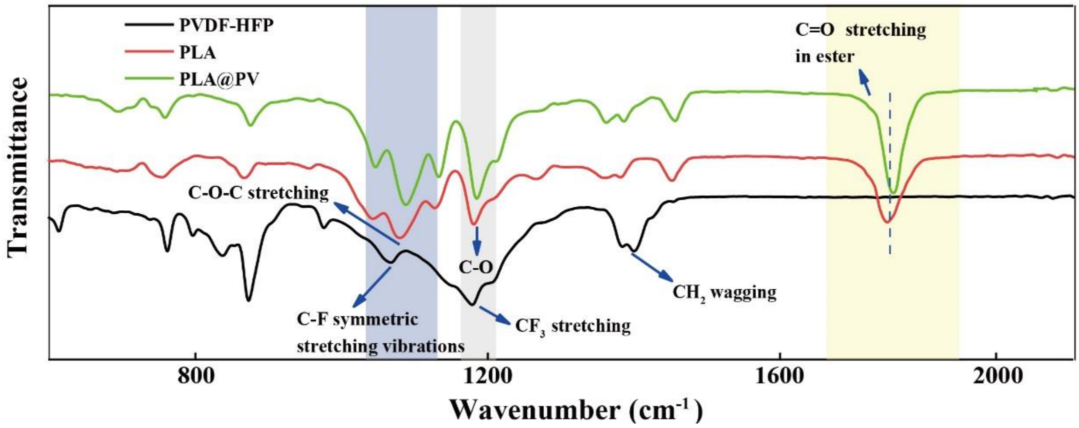

Figure S8. FT-IR spectra of three membranes.

As same as the result of Laput O's research,[1] the peaks observed at 1187 and 1086 cm^{-1} of pure PLA membrane in the present work can be assigned to C–O and C–O–C stretching vibrations. The absorption band at 1750 cm^{-1} in the PLA spectrum is attributed to the stretching vibration of C=O in the ester group of PLA.[2] Moreover, the peaks present at 1060 cm^{-1} (C–F symmetric stretching vibrations),[3] 1172 cm^{-1} (CF_3 stretching) and 1381 cm^{-1} (CH_2 wagging) are assigned to PVDF-HFP.[4, 5] In the case of the 60-PLA@PV membrane, the maintained peak of C=O at 1750 cm^{-1} and the increased intensities of the peaks observed at 1060 and 1172 cm^{-1} compared to the results of two membranes with single component, both indicating a successful synthesis of the biocomposite membrane.

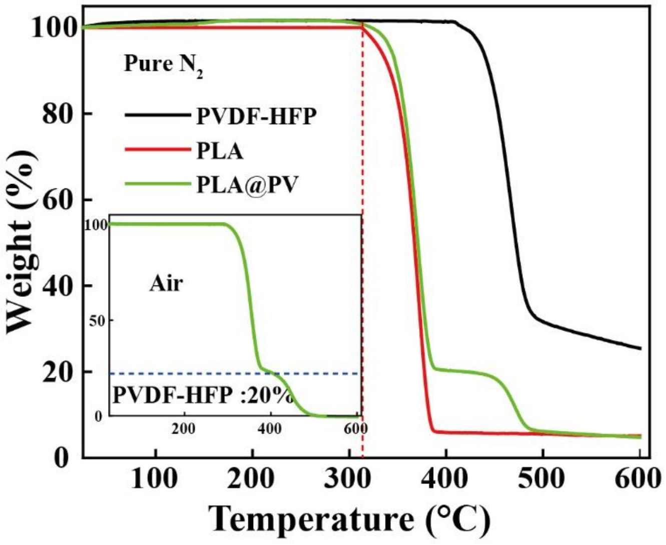

Figure S9. TGA curves of the prepared membranes in N2 and the atmosphere of air.

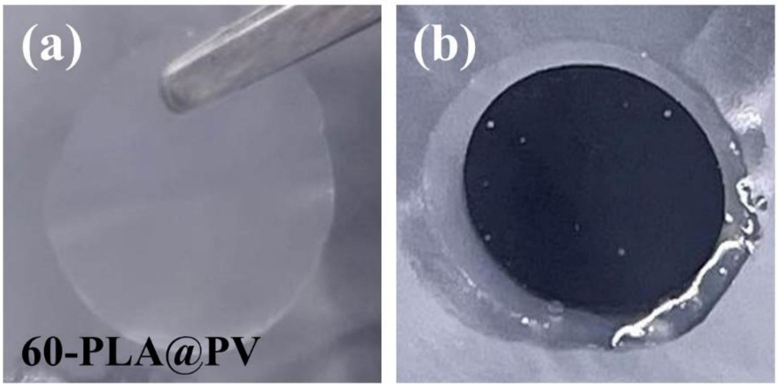

Figure S10. Optical morphologies of two GPE samples after immersion, and interfacial contact between GPEs and electrodes.

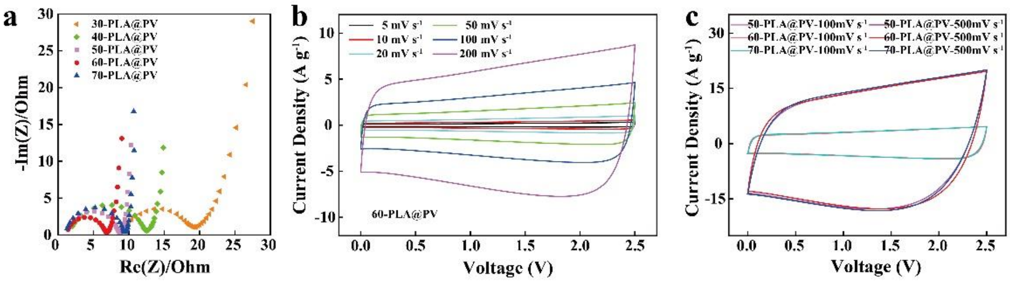

Figure S11. Electrochemical performance of PLA based samples in coin cells: (a) Nyquist plots. (b) and (c) CV curves.

The charge transfer resistance (Rct) calculated according to the diameter of the semicircle are 7.5 Ω, 5.6 Ω and 8.3 Ω for 50-PLA@PV, 60-PLA@PV and 70-PLA@PV, respectively, evidently lower than PVDF-HFP counterpart (11.4 Ω). As a result, lower Rct explains better performances under high current densities due to rapid charge transfer and ionic transport. Figure S11b shows the CV curves of 60-PLA@PV from low (5 mV s^{-1}) to high (200 mV s^{-1}) potential scan rates to testify the electrochemical stability of the prepared sample.

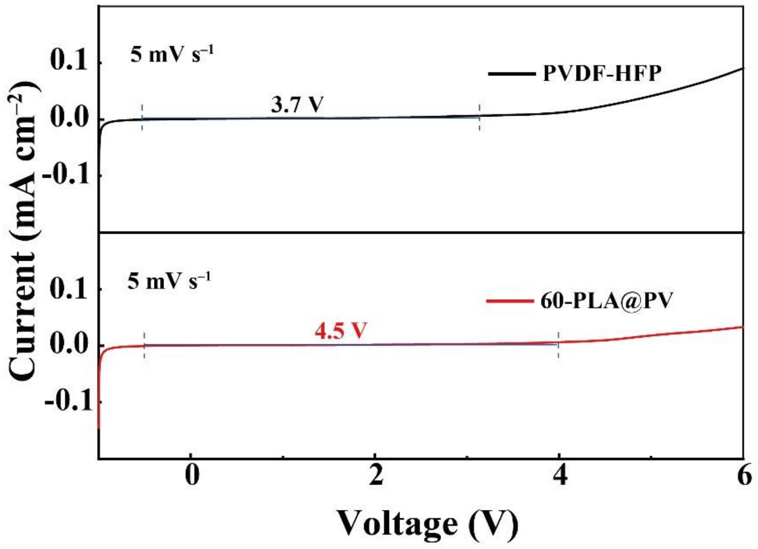

Figure S12. The LSV of SS/GPE/Ag cell (SS: Stainless Steel) (10 mV s^{-1}).

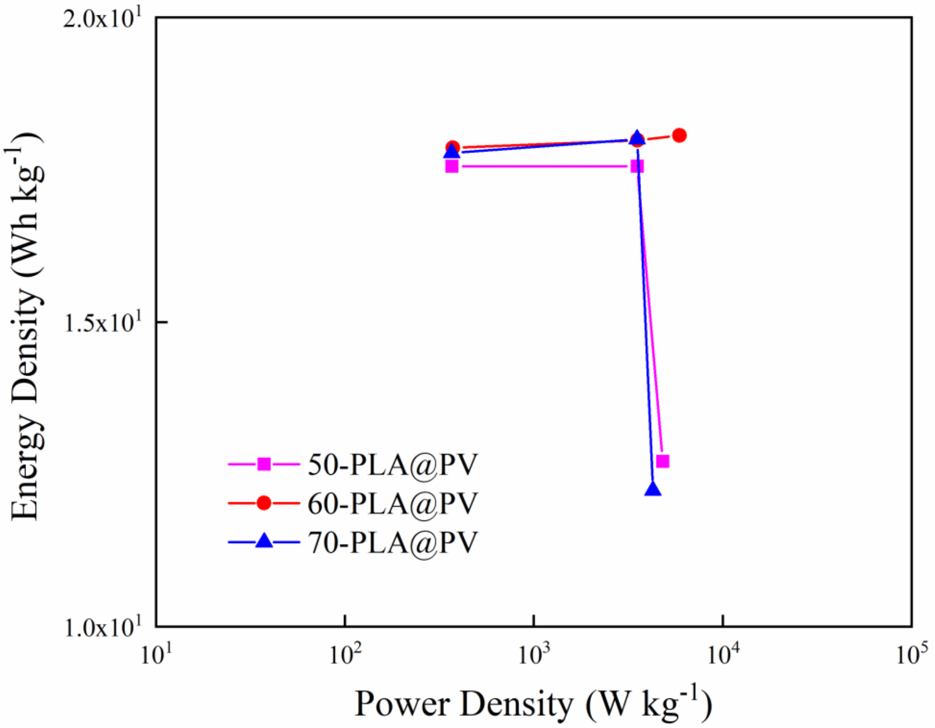

Figure S13. Ragone plot of different PLA based samples in coin cells.

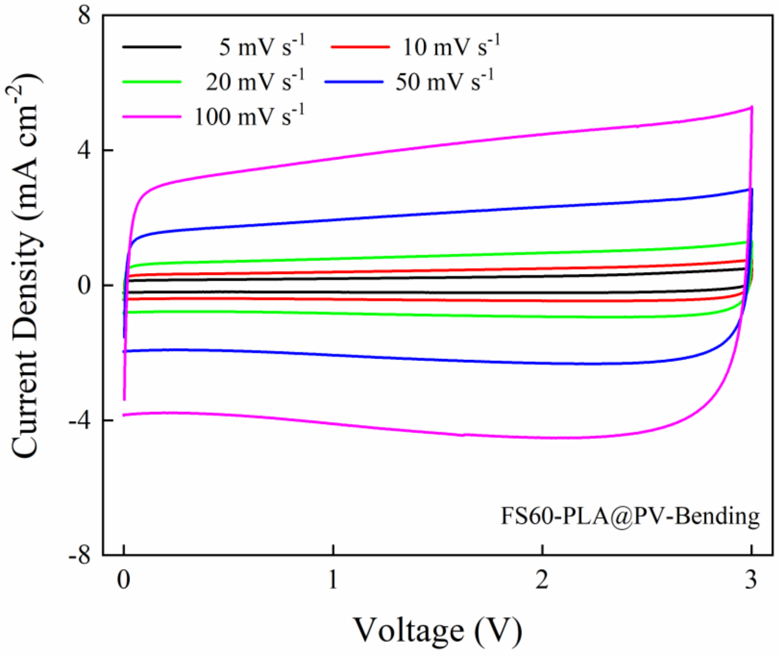

Figure S14. Flexible supercapacitor: CV curves at various scan rates.

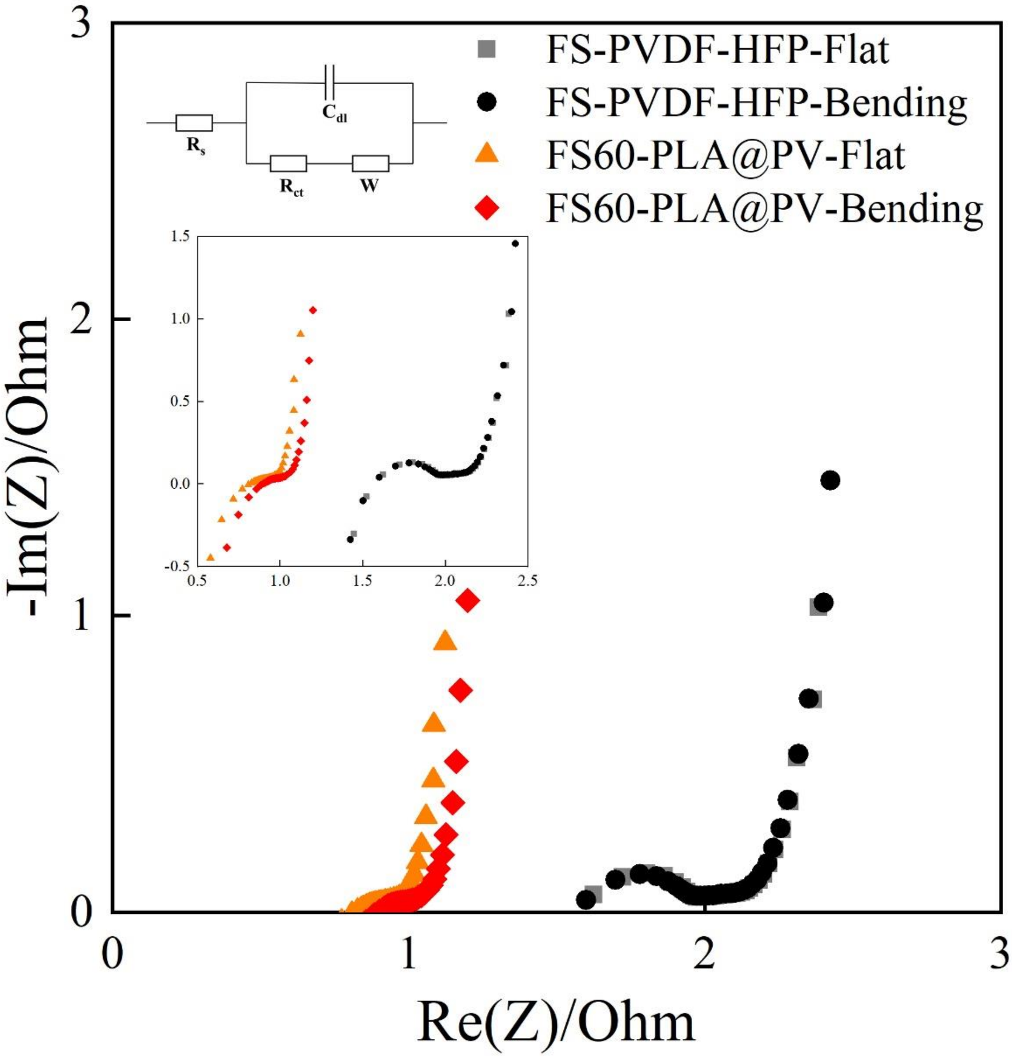

Figure S15. Nyquist plots of FS-PVDF-HFP and FS60-PLA samples under flat and bending states under 3V.

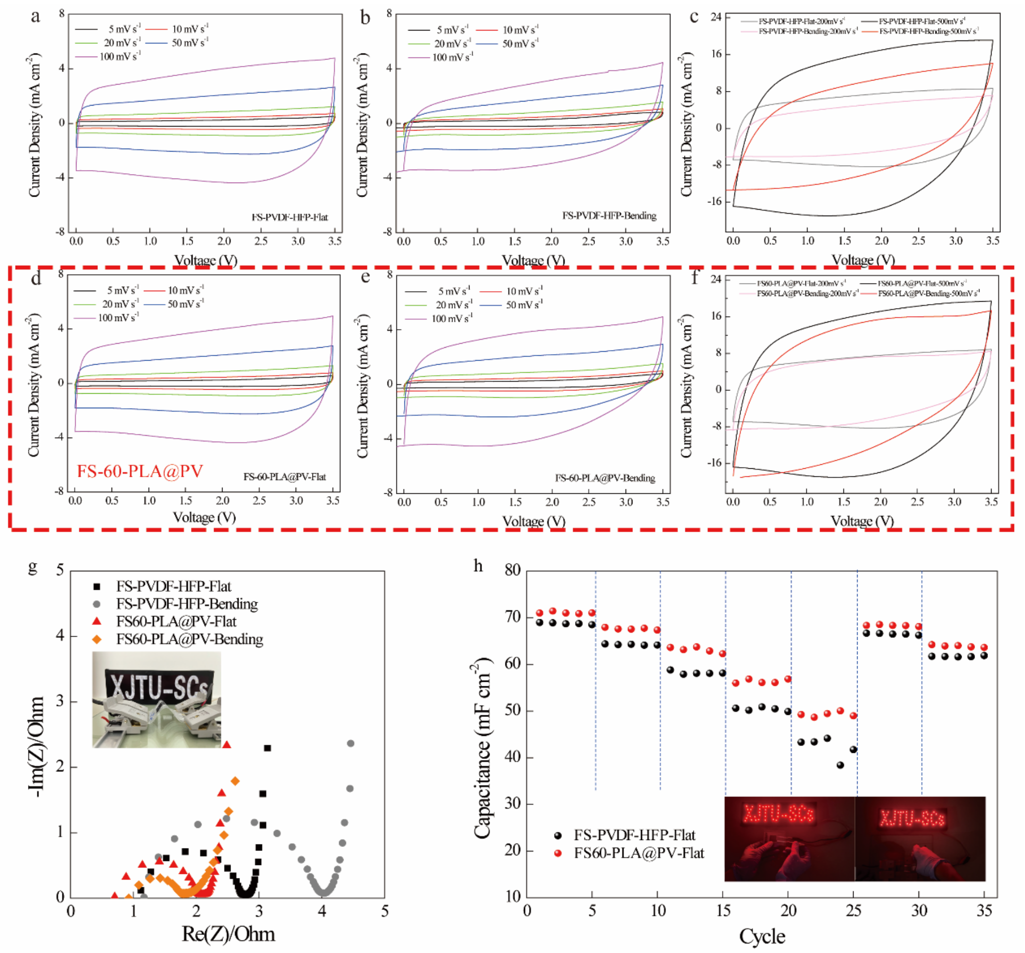

Figure S16. Electrochemical performance of two samples in flexible supercapacitors, (a–f) CV curves of two samples at various scan rates under 3.5 V at flat and bending states, (g) Nyquist plots of FS-PVDF-HFP and FS60-PLA samples under flat and bending states, (h) rate performance of two samples at flat state.

References

1. Laput, O.; Vasenina, I.; Salvadori, M. C.; Savkin, K.; Zuza, D.; Kurzina, I. Low-Temperature Plasma Treatment of Poly(lactic acid) and PLA/HA Composite Material. *J. Mater. Sci.* **2019**, *54*, 11726–11738.
2. Le Mong, A.; Kim, D. Tailor-Made Pore Controlled Poly (arylene ether ketone) Membranes as a Lithium-Ion Battery Separator. *J. Power Sources* **2016**, *304*, 301–310.
3. M. Shanthi, P.; J. Hanumantha, P.; Albuquerque, T.; Gattu, B.; Kumta, P. N. Novel Composite Polymer Electrolytes of PVdF-HFP Derived by Electrospinning with Enhanced Li-Ion Conductivities for Rechargeable Lithium–Sulfur Batteries. *ACS Appl. Energy Mater.* **2018**, *1*, 483–494.
4. Shalu; Singh, V. K.; Singh, R. K. Development of Ion Conducting Polymer Gel Electrolyte Membranes Based on Polymer PVdF-HFP, BMIMTFSI Ionic Liquid and the Li-Salt with Improved Electrical, Thermal and Structural Properties. *J. Mater. Chem. C* **2015**, *3*, 7305–7318.
5. Ahmad, A. L.; Farooqui, U. R.; Hamid, N. A. Effect of Graphene Oxide (GO) on Poly(vinylidene fluoride-hexafluoropropylene) (PVDF- HFP) Polymer Electrolyte Membrane. *Polymer* **2018**, *142*, 330–336.
